# On-Site Pilot-Scale Microalgae Cultivation Using Industrial Wastewater for Bioenergy Production: A Case Study towards Circular Bioeconomy

**DOI:** 10.3390/bioengineering10121339

**Published:** 2023-11-21

**Authors:** Juliana Abraham, Tobi Abimbola, Washington J. Braida, Amalia Terracciano, Tsan-Liang Su, Christos Christodoulatos, Agamemnon Koutsospyros, Abhishek RoyChowdhury, Benjamin Smolinski, Adeniyi Lawal

**Affiliations:** 1 Center for Environmental Systems, Stevens Institute of Technology, Hoboken, NJ 07030, USA; wbraida@stevens.edu (W.J.B.); aterrac1@stevens.edu (A.T.); t1su@stevens.edu (T.-L.S.); christod@stevens.edu (C.C.); akoutsos@stevens.edu (A.K.); aroychowdhury@navajotech.edu (A.R.); 2 New Jersey Center for MiFantao Kongcrochemical Systems, Department of Chemical Engineering and Materials Science, Stevens Institute of Technology, Hoboken, NJ 07030, USAalawal@stevens.edu (A.L.); 3 Combat Capabilities Development Command—Armaments Center (DEVCOM—AC), Picatinny Arsenal, Dover, NJ 07806, USA; benjamin.l.smolinski.civ@army.mil

**Keywords:** on-site pilot scale, industrial wastewater valorization, carbon capture, areal biomass productivity, energy potential, sustainability

## Abstract

This case study assesses the valorization of industrial wastewater streams for bioenergy generation in an industrial munition facility. On-site pilot-scale demonstrations were performed to investigate the feasibility of algal growth in the target wastewater on a larger outdoor scale. An exploratory field study followed by an optimized one were carried out using two 1000 L open raceway ponds deployed within a greenhouse at an industrial munition facility. An online system allowed for constant monitoring of operational parameters such as temperature, pH, light intensity, and dissolved oxygen within the ponds. The original algal seed evolved into an open-air resilient consortium of green microalgae and cyanobacteria that were identified and characterized successfully. Weekly measurements of the level of nutrients in pond liquors were performed along with the determination of the algal biomass to quantitatively evaluate growth yields. After harvesting algae from the ponds, the biomass was concentrated and evaluated for oil content and biochemical methane potential (BMP) to provide an estimate of the algae-based energy production. Additionally, the correlation among biomass, culturing conditions, oil content, and BMP was evaluated. The higher average areal biomass productivity achieved during the summer months was 23.9 ± 0.9 g/m^2^d, with a BMP of 350 scc/gVS. An oil content of 22 wt.% was observed during operation under low nitrogen loads. Furthermore, a technoeconomic analysis and life cycle assessment demonstrated the viability of the proposed wastewater valorization scenario and aided in optimizing process performance towards further scale-up.

## 1. Introduction

Over the last few decades, the pursuit of renewable energy resources has followed different approaches. The conversion of solar into chemical energy, known as photosynthesis, has been explored with the aim of producing biomass with high energy potential. It has been established that, compared to terrestrial plant biomass, algae can produce the highest areal biomass yields, thus making them a preferred substrate for renewable biofuel applications employing a variety of municipal, saline, or industrial wastewater [1,2,3]. In addition, photosynthetic microorganisms, like microalgae and cyanobacteria, have two important advantages: (1) They grow fast and (2) they can be cultured in non-arable lands. Hence, they do not compete with food production and could minimize the need for freshwater resources [4]. Moreover, several types of bioenergy can be produced from algae biomass via chemical, biochemical, and/or thermochemical conversion into biogas, biodiesel, green diesel, sustainable aviation fuel (SAF), hydrogen gas (H_2_), and other biofuels [5]. To date, several studies have shown substantial benefits in the integration of wastewater-grown microalgae-based biofuel production [6,7,8,9,10].

The production of algal biomass for food, biofuels, and other commercial products dates from the early 1950s and has been documented in more than 17,000 publications worldwide [11,12]. Among several reactor configurations used for this purpose, open pond systems are the most widely used. The major advantages of open pond systems are the low capital and operating costs as well as the low energy requirements for culture mixing. Conversely, open pond reactors need large areas to scale up and are susceptible to weather conditions and contamination. Yet, sustainability studies show that efforts should be focused on enhancing biomass productivity, valorizing all products obtained from biomass and improving the integration of various industrial nutrient sources from waste streams to lower the cost of operations [13,14]. 

Diverse options may be considered for maximizing the recovery of bioenergy from algal biomass within the framework of a net-zero conversion process. In this scenario, anaerobic digestion (AD) is one of the most promising and feasible technologies for the valorization of algae biomass cultures both as a product (i.e., raw) and after lipid extraction (i.e., residue post extraction) [15]. The process is particularly attractive due to the production of biogas, a mixture of methane and carbon dioxide with traces of volatile organics and hydrogen sulfide. Due to the biomethane content and easier compression storage, biogas is characterized by its superiority in energy conservation and emissions reduction. Biomethane represents a renewable fuel source that can be combusted to generate heat and electricity for residential, commercial, industrial, and transportation purposes [16,17]. The biochemical methane potential (BMP), defined as the total methane yield per unit of substrate added, represents a key parameter for assessing the suitability of AD feedstocks [18]. The incorporation of AD is considered critical for both economics and sustainability reasons since this technology provides a main route for recycling nutrients to the algae cultivation process [19]. 

The reuse and recycling of nutrient resources are particularly important for minimizing the environmental footprint of the biofuel production chain. In that respect, the processes considered in this study align with general circular bioeconomy approaches, in which the production of renewable biological resources and their conversion into value-added products is highly promoted [20,21]. For instance, atmospheric nitrogen fixation for ammonia production (via the Haber–Bosch process) and its subsequent conversion to other nitrogen species of interest (i.e., nitrate salts, nitric acid, etc.) consume valuable resources and generate products that require treatment, exerting additional resource demand and further increasing the environmental footprint. By utilizing nitrogen-rich waste streams in the current study, a portion of the nitrogen species can be reclaimed and converted into biomass, generating a valuable resource (i.e., for bioenergy or other uses), along with savings in denitrification treatment costs.

In this case study, the technical and economic feasibility of growing algae feedstocks for biofuel production was assessed using two 1000 L open raceway ponds and industrial wastewater in an outdoor setting at an industrial munitions facility located in the continental US. The study falls under the facility’s efforts to reduce its environmental and energy footprint by utilizing nutrient-rich wastewaters to produce useful bio-stocks. The study focuses on the improvement of algal biomass productivity and bioenergy potential. The two most researched pathways to generate energy from algae, oil, and biogas were assessed. Moreover, to complement the assessment of the experimental approach, a techno-economic analysis (TEA) and life cycle assessment (LCA) for a 100 ha algae farm were performed with the data obtained in this study. On one hand, TEA establishes the capital and operating cost profile to determine the potential economic viability of the selected conversion process towards commercial feasibility. On the other hand, LCA evaluates the potential environmental and social impacts associated with a product throughout its life cycle from raw material extraction to disposal [22]. The global warming potential over 100 years (GWP_100_) per mega joule of energy produced was used as an impact category. To the best of our knowledge, this is the first on-site study assessing the valorization of this type of industrial nitrogen-rich wastewater for bioenergy generation purposes.

## 2. Materials and Methods

### 2.1. Wastewater Characterization and Aquatic Toxicity Assessment

The wastewater streams used for this study were an industrial wastewater plant influent (IWWPI) and an industrially generated ammonium nitrate (AN) filtered solution (~65% ammonium nitrate content). Both streams were thoroughly characterized and tested for aquatic toxicity to microalgae, as described in previous work [23,24]. Wastewater characterization was performed by analyzing total carbon (TC) and total organic carbon (TOC) using a Teledyne Tekmar Fusion TOC analyzer (Mason, OH, USA). The concentration of ammonium; mono-, di-, and trimethylammonium; nitrates; nitrites; phosphates; and sulfates was determined by Dionex Aquion and Integrion ion chromatography (Madison, OH, USA) equipped with IonPac^®^ CS16 (4 mm × 250 mm, Dionex, Sunnyvale, CA, USA) and AS18 (2 mm × 250 mm, Dionex) columns for cations and anions, respectively. The presence and concentration of energetic compounds (ECs) were determined with a mass spectrometer (Quattro Ultima, Waters, MA, USA) equipped with an electrospray ion source (ESI-MS) and by an Agilent 1260 Infinity HPLC (Wilmington, DE, USA) following the US EPA Standard Method 8330B [25]. The pH and electrical conductivity were measured with an Oakton^®^ PC 700 m and their respective probes. Aquatic microalgal toxicological assessment was evaluated by the OECD protocol adjusted to 24-well microplates. The inhibition of the growth rate (*Inh*.) was calculated by comparing the specific growth rate (*µ*) for each sample/concentration tested (*µs*) to the control (*µc*) using the following equation:

(1) $$Inh.\left(\%\right)=\frac{\;\left(\mu c-\mu s\right)}{\mu c\;}\times 100\;\;\;\;\;\;\;\;\;\;\;\;\;\;$$

### 2.2. Open Raceway Ponds and Operating Conditions

**Figure 1:**
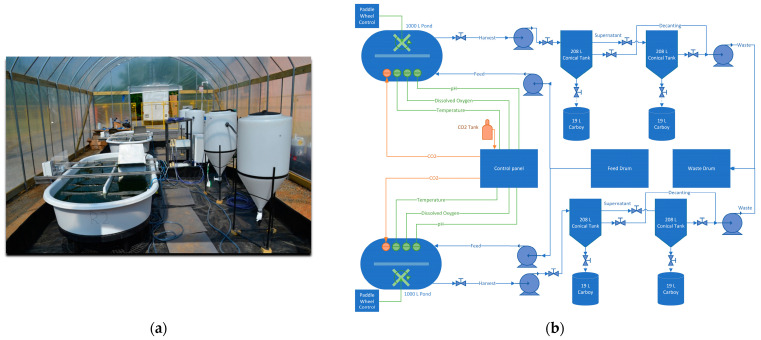
Culture of microalgae using industrial wastewater: photo (**a**) and schematic of algae reactor pilot system layout at the plant greenhouse (**b**).

The study was conducted using two open raceway ponds with a 1000 L working volume and a surface area of 3.4 m^2^ each (MicroBio Engineering, San Luis Obispo, CA, USA), located within a greenhouse at the munitions facility. Data from continuous monitoring of pH, temperature, and dissolved oxygen (DO) were logged and stored hourly using an APEX-Fusion controller (Neptune systems, Morgan Hill, CA, USA). Figure 1 illustrates the layout of the field pilot-scale system. Photosynthetically active radiation (PAR) was measured using three quantum sensors. Two LI-193 spherical quantum sensors measured the PAR underwater inside of each reactor, and one LI-192 sensor was installed to measure PAR in all directions outside of the reactors. Direct digital readout and data logging from the three LI-COR sensors were provided by a LI-1500 Light Sensor Logger (LI-COR, Lincoln, NE, USA).

Two sets of experiments were conducted during the periods of May–October 2019 and May–July 2021, namely, an exploratory run (ER) and an optimized run (OR), with the pilot-scale reactors operated in sequential batch mode. Samples were collected from both reactors, R1 and R2, on a weekly basis before and after harvesting to determine nutrient concentration, fluorescence levels, and ash free dry weight (AFDW). Filtered dechlorinated water was added daily to each reactor to compensate for evaporation losses.

The initial algal seed for the pilot-scale reactors was generated using two 100 L open raceway ponds operated at Stevens Institute of Technology (SIT). A mixture of wastewater streams resulting from AN solution highly diluted in IWWPI and supplemented with macro and micronutrients, as described for the BG-11 medium [23], was used. Depending on the amount of N utilized (Table 1), two different approaches were applied for pH control and carbon addition: CO_2_ bubbling was enabled when the pH increased above 7.5, and sodium carbonate was used to elevate the pH and carbon content in the bulk liquor when the pH dropped below 5.5. 

During harvest, 400 L from each raceway pond were transferred into conical settling tanks (Figure 1), allowing the algae to settle for 3–3.5 h and recovering the concentrated biomass. Subsequently, another 400 L of liquor were pumped out of the conical tanks and allowed to settle; the concentrated algal mass was collected while the supernatant was disposed of in the industrial wastewater treatment plant during the ER or partially recycled (50 %) into the ponds during the OR. 

For a few selected harvests, ferric chloride solution (2–4 L/tank, 4000 mg/L) was used to enhance the settling process. The biomass collected at each harvest was shipped to SIT for further analysis. 

### 2.3. Sample Analysis 

Weekly measurements of biomass growth and nutrients were performed over the testing period. Cell density was determined using a microplate reader (Cytation 3, Biotek, Winooski, VT, USA) with a chlorophyll filter to measure fluorescence at 440–685 nm excitation–emission, whereas dry mass, expressed as ash-free dry weight (AFDW), was determined by measuring the total suspended solids and volatile solids (TSS and VSS, respectively, g/L) following the standard method APHA/AWWA/WEF 2540 D [26]. Algal microflora were examined and characterized under a compound light microscope at Fantao Kong, 100×, and 400× magnification. Samples were scanned for algal taxa, whose relative abundance was recorded. Micrographs were also captured using a digital microscope (Biotek, VT, USA) with bright-field and fluorescence (chlorophyll filter) modes. In addition, shotgun metagenomic sequencing using Illumina HiseqX10 was performed to gain insight into the taxonomy of the polyculture developed on site and used for the study. Macronutrients (C, N, P) were determined as described in Section 2.1. 

Areal biomass productivity was calculated according to [27]. Briefly, the harvest yield areal productivity was calculated by considering the amount of algal biomass harvested from each of the two ponds (AFDW) multiplied by the volume harvested and divided by the surface area of the ponds (3.4 m^2^) and the cultivation time (days of culture). The productivity for each pond was then averaged to obtain the average areal productivity, expressed as g AFDW per m^2^ per day (g AFDW/m^2^d). Algal slurry samples from reactors R1 and R2 were received at SIT for additional dewatering by centrifugation as described in [28]. The total solids (TS) and volatile solids (VS) of each sample were determined following the National Renewable Energy Laboratory (NREL) TS and VS analytical procedures [29]. Concentrated algal pastes were preserved in the refrigerator to prevent physicochemical changes in the algal biomass. Elemental analysis of inoculum and biomass obtained by selected harvests was performed using a CHN elemental analyzer. Furthermore, these samples were mixed with acetonitrile and sonicated for 30 min to extract ECs that might potentially be adsorbed on algae biomass. Subsequently, the extractant was analyzed for energetic compounds by MS and HPLC as described in Section 2.1.

### 2.4. Extraction and Quantitative Analysis of Algal Oil

A detailed description of the oil extraction apparatus can be found in [30]. For this study, a constant amount of ethanol (100 g solvent), 30 g of algal paste at ~7.5 wt.% TS, and an extraction time of 3 h were used. The quantification of the crude oil extract was performed subsequently as described in the referenced work. 

### 2.5. Anaerobic Digestion (AD) Configuration and Biogas Analysis 

The TS of algae used for AD was about 2.5% (mass). Digested sludge (DS) containing a mixed culture of microbes was used as an inoculum. The DS was received from Bergen County Utility Authority (BCUA, Little Ferry, NJ, USA). The DS was degassed as recommended by [31] prior to feed initiation. The AD system consisted of a 245 mL microcosm bottle equipped with a rubber stopper to prevent the leakage of produced biogas under the maximum proof pressure tested. Both negative (only 90 g of DS) and positive (90 g of DS with 1 g glucose) controls were prepared in addition to other samples tested. The working volume of the microcosm bottle was 90 mL, and the produced biogas occupied the remaining 155 mL headspace. The samples were prepared by mixing the DS with the algae such that the volatile solid (VS) ratio of DS to algae was 2:1. The AD test was performed in a controlled temperature shaker (37 °C) at a speed of 150 rpm. Before placing the samples in the shaker, nitrogen gas was used to purge each sample for at least 5 min to create anaerobic conditions. All AD experiments for the algal samples and the controls were performed in duplicate.

Initial headspace pressure was 0 psig at the start of the AD experiment and subsequently increased depending on the amount of biogas produced as the reactions progressed. The change in headspace pressure, recorded daily by a manometer, was used to obtain the VSTD (volume of biogas produced at STP) using the formula below:

(2) $$\mathrm{Vstd}=\frac{\left(\Delta P\;\times \mathrm{Vi}\;\times \;\mathrm{Tstd}\right)\;\;\;}{\left(\;\mathrm{Pstd}\;\times \;\mathrm{Ti}\;\right)}$$

where ΔP = gauge pressure reading on the manometer (psig), Ti = 308 K (35 °C, temperature of the AD), Vi = headspace of the microcosm (~155 mL), Pstd = 14.696 psi (standard pressure), and Tstd = 273 K (0 °C standard temperature).

The composition of the biogas produced from all AD samples was determined by a Shimadzu GC-14B-TCD equipped with Plot Q and Mole sieve columns in series. Argon was used as a carrier gas and the GC oven was programmed isothermally at 30 °C for 20 min. The detector temperature was set at 150 °C and the injector temperature was set at 220 °C.

Subsequently, the amount of biomethane produced (standard cubic centimeters, scc) was calculated by multiplying VSTD with the mol % of CH_4_ obtained from the GC. The biochemical methane potential (BMP) was reported as a cumulative measurement (in scc) of biomethane produced per gram of volatile solids (VS) of the initial algal biomass fed. The net biomethane generated (BM) was obtained by subtracting the biomethane produced by the added DS inoculum (negative control) from the cumulative biomethane (BM) produced by the digested sample. A positive control was investigated to assess the accuracy of the test.

## 3. Results and Discussion

### 3.1. Wastewater Analysis

An extensive characterization of the industrial wastewater streams used in this study was first carried out. Table 2 lists some of the characteristics of the two wastewater streams used: industrial wastewater plant influent (IWWPI) and industrially generated ammonium nitrate filtered solution (AN). The latter contains a high concentration of nitrogen (N) in the form of ammonium and nitrate; both ammonia and nitrates are macronutrients that algae can use to grow to a certain threshold. In addition, ammonia can be easily assimilated since microorganisms can use it directly without any reduction or energy demands, as required for nitrate assimilation [32,33].

Table 3 shows the different AN concentrations tested, along with the calculated growth rate (µ) and the % of growth rate inhibition. In these tests, all of the nutrients were added in excess to avoid growth inhibition due to lack of nutrients, with the exception of N provided by AN. Results from tests with different nitrogen concentrations demonstrated that AN can be used as a sole source of nitrogen for growing algae biomass without significant toxicity up to approximately 750 mg N/L (Figure S1). In fact, concentrations of total nitrogen higher than 1000 mg N/L resulted in inhibition of growth, as evident by the absence of cell chlorophyll and green color development. From this assessment, AN solution was used after dilution with IWWPI. The amount of AN added initially, and after every harvest, upon refill of the raceway reactors fell within the first four columns of serial dilutions in Table 3, resulting in a total nitrogen concentration in the order of 50–200 mg/L (depending on the tested low/high N range, as described in Table 1). The level of N was determined based on the toxicity results and the amount of nitrogen remaining in the supernatant liquor after the algae settled. The rationale was based on minimizing the amount of nitrogen that is recycled to the IWWTP for treatment and discharge. 

Similarly, a high amount of ammonia nitrogen from dairy farm wastewater [34] and digested piggery wastewater [35] streams have been valorized as a source of algal growth; however, to date, there is no evidence of the utilization of this nitrogen-rich wastewater from munition facilities.

### 3.2. Culture Conditions and Areal Biomass Productivity

#### 3.2.1. Seeding the Reactors: Developing a Resilient Consortium

For this study, an initial seed of freshwater microalga *S. obliquus* was acclimated to grow in the selected wastewater mixture under controlled conditions (i.e., 25 °C, continuous 120 rpm agitation, 68 µmol photons/m^2^s with a 14:10 h light:dark photoperiod) in 5 L flasks, which later served as an inoculum for the 100 L open pond reactors. Once the 100 L reactors achieved a high concentration of biomass (~1 g/L), the algal liquor was transported to the industrial facility and used to seed the on-site 1000 L reactors. As stated in our previous laboratory and indoor pilot work [23,28,30], *S. obliquus* strain was originally selected due to its high growth rate, ability to grow in wastewater streams, and improved lipid content when cultured in N-depleted media. However, in this study, the scaling up to an outdoor environment showed a rapid development of various species, opening the possibility of developing a polyculture more adaptable to the local environmental conditions. In open pond systems, the benefits of polycultures (more than 4 or 6 species) over monocultures include improved biomass productivity and resilience to disturbances by predators or adverse environmental conditions [36].

**Figure 2:**
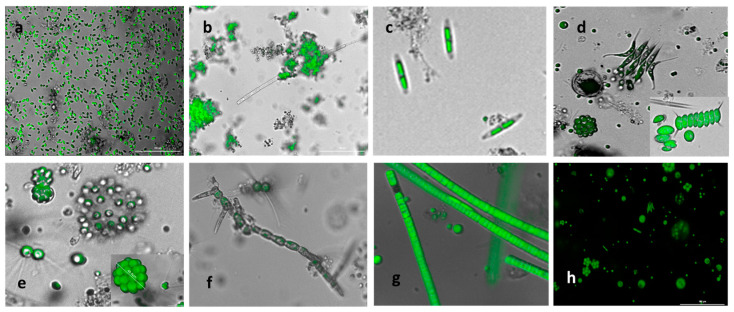
Microscopy pictures showing the shift from the initial predominant *S. obliquus* culture to a more resilient consortium (**a**) initial inoculum cultivated at SIT, mainly *S. obliquus* grown in IWWPI and AN wastewater; (**b**) microalgae tend to form clusters and aggregate with fibers and particles present in the wastewater; (**c**) presence of freshwater diatoms; (**d**) *Desmodesmus* sp., *Coelastrum* sp., and *Scenedesmus* sp. (inset); (**e**) *Dyctosphaerium* sp., *Micractinium* sp., and *Coelastrum* sp. (inset); (**f**) *Stigeoclonium* sp.; (**g**) *Ulothrix* sp.; and (**h**) assorted population in the pond. The pictures are a merging between the bright field and the fluorescence image (400x); 2 h: only fluorescence.

An assembly of microscopy images that depict the microbial population in the 1000 L outdoor open ponds is shown in Figure 2. The initial predominant culture of *S. obliquus* (with characteristic rice shape, Figure 2a) evolved naturally to a diverse culture of microorganisms. Most of the microorganisms developed were green freshwater microalgae and cyanobacteria. Most likely, the new species were introduced into the system via the wastewater feed streams and the atmosphere. Furthermore, the system’s microbial ecology evolved in response to the prevailing environmental and operational conditions, such as temperature, pH, solar radiation, and feed composition. Clusters of green microalgae developed due to the presence of fibers and other particles present in the wastewater (Figure 2b). Additionally, these clusters could have developed because of strong grazing pressure to the initial monoculture [37]. 

The development of diverse freshwater green microalgae and cyanobacteria observed included *Ankistrodesmus*; pennate diatoms (Figure 2c); spiny microalgae with the formation of typical protective eight-celled colonies from the *Scenesmaceae* family like *Desmodesmus*, *Scenedesmus, Tetradesmus*, and *Coelastrum (*Figure 2d,e); and round microalgae from the *Chlorellaceae* family such as *Chrorella*, *Chlorococcum*, *Micractinium*, and *Dictyosphaerium* (Figure 2d,e). Filamentous algae identified as *Stigeoclonium* (Figure 2f) and *Ulothrix* (Figure 2g) also developed in the ponds, as well as the cyanobacteria *Aphanizomenon*, *Nostoc*, and *Calothrix*, amongst others. A metagenomic analysis confirmed the species identified by microscopy, as shown in Figure S2. This consortium prevailed in both experimental runs.

#### 3.2.2. Exploratory Run (ER): Operating Conditions and Performance

The exploratory run was performed from spring to fall of 2019 using the industrial wastewater streams characterized in Section 3.1 and the autochthonous polyculture developed on site described in Section 3.2.1. The conditions of this study are described in Table 1. Over this period, operational parameters were monitored as described in Section 2.1. In addition, two N concentrations were used to evaluate biomass productivity as well as bioenergy production (Table 1). The water temperature of the ponds varied significantly over the course of the day–night cycle; moreover, the temperature changed from the first month of operation, with the temperature increasing several degrees due to the transition from spring to summer and then gradually decreasing going from summer to fall (Table 4). Overall, the cultures were grown under an average temperature within the optimum range for algae growth (i.e., 20–30 °C) [38]. 

**Figure 3:**
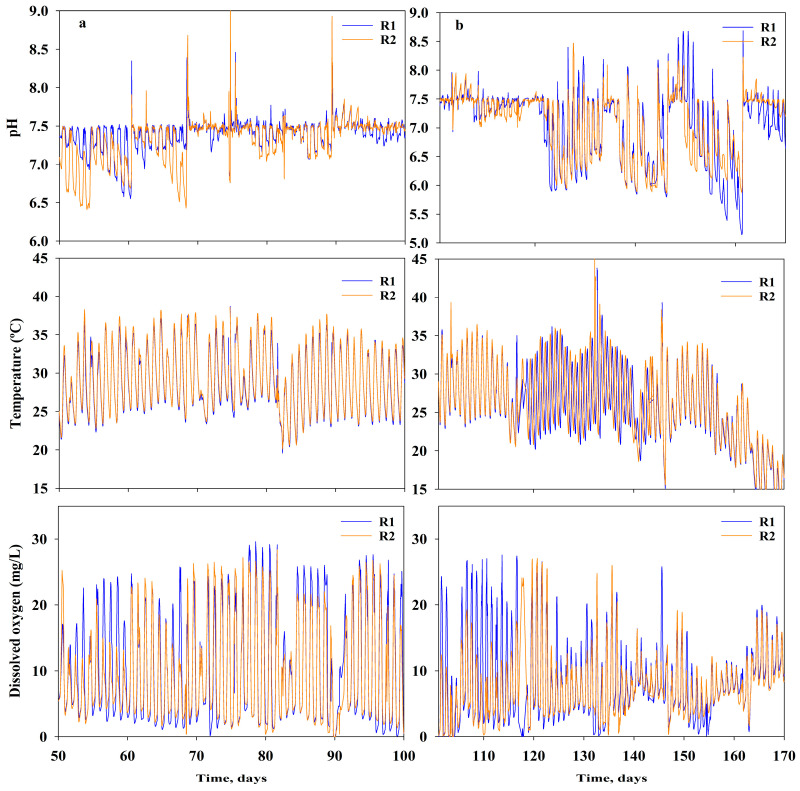
Online measurement of pondwater pH, temperature, and DO at each of the reactors during the exploratory run under (**a**) low N conditions (left side) and (**b**) higher N loads (right side).

Furthermore, DO varied from 0 to 29.6 mg/L and 28.4 mg/L for R1 and R2, respectively. The highest DO values measured corresponded to DO levels during daylight hours when photosynthetic activity was high. Conversely, the low DO values were recorded at nighttime, when photosynthesis was absent and natural reaeration was not capable of satisfying the oxygen demand required by biomass respiration. Figure 3a shows the pH, T, and DO during the testing of low N concentrations, whereas Figure 3b shows the same parameters when testing higher N loads.

The pH was maintained within 6.50–7.50 by bubbling CO_2_ when the pH was higher than 7.50. Conversely, Na_2_CO_3_ was added when the pH was lower than 6.50; this approach was needed with the use of higher concentrations of AN (i.e., ER—high N feed, from 8 H to 13 H). In fact, the consumption of ammonia caused a decrease in pH, making it necessary to add alkalinity to maintain satisfactory productivity yields (Figure 3b). Eustance et al. [39] reported similar results at laboratory scale; they observed an extreme shift in pH when the strains were grown at high ammonium concentrations (i.e., 2.94 mM ammonium chloride) in unbuffered medium, which triggered growth inhibition and chlorophyll degradation. Furthermore, the results from our previous work [40] showed that mineral carbonates such as sodium carbonate (Na_2_CO_3_) or MgCO_3_.3H_2_O (nesquehonite) can buffer the pH well and maintain it within acceptable algae physiological values. 

Biomass growth over time was evaluated by measuring fluorescence (fluorescence units, FU) and ash-free dry weight (AFDW, in g/L). Fluorescence measurements were performed as a rapid estimate of healthy biomass accumulation in the ponds since this measurement is linked to chlorophyll. A linear correlation between AFDW and FU was found (Figure S3), suggesting that even though dry biomass is an essential parameter for areal productivity evaluation, the measurement of FU is an additional rapid way to monitor the culture’s growth, particularly on site, when sometimes decisions need to be made in a short amount of time. 

**Figure 4:**
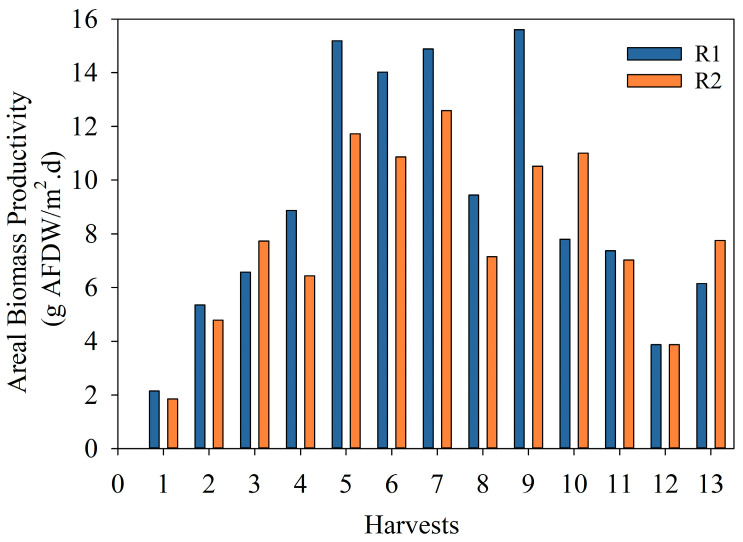
Areal biomass productivity (g AFDW/m^2^d) of microalgae in the two 1000 L raceway reactors in the exploratory run.

Figure 4 shows the areal biomass productivity calculated for each reactor and each harvest during the exploratory run. The average areal biomass productivity was 15 and 10 g/m^2^d for R1 and R2, respectively, over the summer period, dropping to 8.16 and 8.04 g/m^2^d for R1 and R2, respectively, during fall. Overall, the areal productivity observed for each reactor per season was reproducible. These values were similar to the ones found in the literature [36,41,42]. However, the difference between R1 and R2 for the summer period could be related to the reactor’s orientation (not in parallel due to space constraints) and the light intensity received during the day. The ER aimed to assess process feasibility and identify site-specific operational limitations, biomass productivity, and related seasonal changes. Hence, from the information gathered, required changes were implemented in the optimized run to increase biomass productivity and bioenergy potential. 

#### 3.2.3. Optimized Run (OR): Improving Operating Conditions

**Figure 5:**
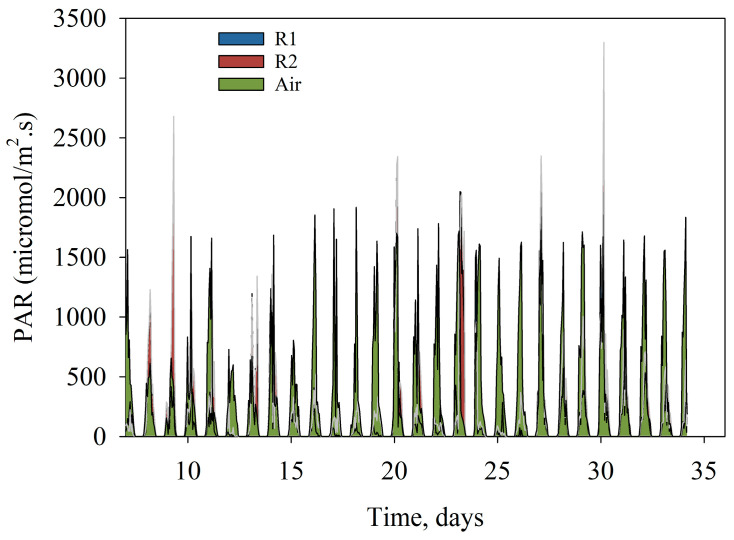
Photosynthetically active radiation (PAR, in µmol/m^2^s) in air and inside the raceway ponds. The *x*-axis represents the days from reactor setup.

The optimized run was performed in late spring to early summer of 2021 (Table 1), during which lower N amounts were supplied from AN wastewater alone, and the harvesting frequency was reduced to between 3–5 days. For this run, the photosynthetic active radiation or quantum radiation (PAR) was also measured (Figure 5); the PAR measure inside each reactor was similar, although R2 showed higher PAR readings, likely due to the reactor orientation. Additional PAR readings were obtained from the air-exposed sensor, which measured the light intensity reaching the surface of the reactors; the highest intensity measured and calculated from the area under the curve was 1418 µmol/m^2^s, which was threefold higher than the minimum reading of 411 µmol/m^2^s. High PAR readings occurred around harvests 3, 4, and 5, which also matched the highest biomass productivity. 

**Figure 6:**
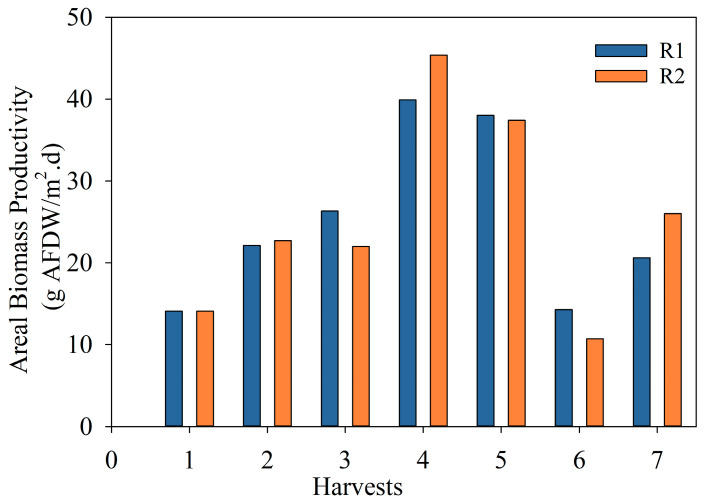
Areal biomass productivity (g AFDW/m^2^d) of microalgae in the two 1000 L raceway reactors in the optimized run.

Figure 6 shows the areal biomass productivity for the OR after each harvest. The average areal biomass productivity for the duration of this run was 23.3 and 24.5 g/m^2^d for R1 and R2, respectively. The average areal productivity for the entire run was 23.9 ± 0.9 g/m^2^d. 

Overall, the productivity increased twofold compared to the ER (i.e., an average of 12 g/m^2^d in the same period) by controlling the nutrient level from N species and increasing the harvesting frequency. As this study was only performed during late spring–early summer, areal productivity values should be adjusted, taking into consideration seasonal variations. A predominance of filamentous photosynthetic microorganisms was observed due to the higher frequency of harvests compared to that of the previous run. 

### 3.3. Biomass Harvested from ER and OR

Elemental analysis (CHN) results for the ER show that the carbon content in the initial biomass increased from 38% to 47% and 49% at the eighth harvest (8H) for R1 and R2, respectively. Thereafter, the carbon content in the biomass decreased to 38% and 36% for R1 and R2, respectively. This trend correlates well with the increase in N in the feed from harvests 8H to 13H. The observed biomass increase of more than 20% of carbon content was also reflected in the oil content from the same harvests (Section 3.4). Several studies observed increments in the carbon and lipid contents for microalgae cultured under nitrogen limitations [28,43,44].

Furthermore, for the OR, a similar trend for carbon content was observed (the carbon content of 38% in the initial biomass increased to 46% and 47% for R1 and R2, respectively). Although a similar C composition was observed in both runs (ER—low N and OR), the higher harvesting frequency adopted in the OR seemed to promote the increase in biomass productivity, to the detriment of the biomass’s oil content (Section 3.4). Such results are consistent with those reported in the literature [45,46].

Finally, the results from solvent biomass extraction demonstrate that no energetic compounds were present in the algae biomass harvested, eliminating any potential interference on the downstream algae processing. Figure S4 shows the mass spectrometry scans for several acetonitrile extracts, in which no peaks were observed for any of the energetic materials of potential concern.

### 3.4. Oil Content Obtained from ER and OR

An additional goal of the current study was to evaluate the oil content within the wastewater-grown biomass to estimate its energy potential. 

**Figure 7:**
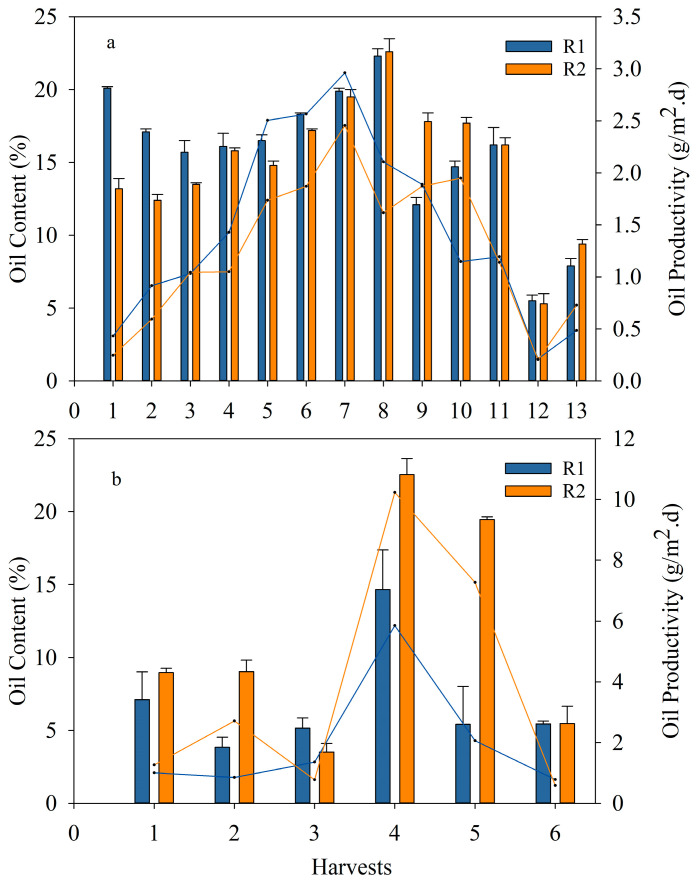
Oil content (bars) and oil productivity (lines) obtained in R1 and R2; (**a**) exploratory run for each harvesting period with limited N (up to 8H) and N in excess (from 9H to 13H) and (**b**) optimized run. The data shown are the mean ± error of at least two measurements per reactor per harvest.

Initially, the oil content of algae grown in the 100 L raceways ponds (seed) was assessed using the extraction method presented in Section 2.4, with an oil content of *S. obliquus* of 21.7 ± 0.5%. During the ER, the oil content was extracted from the harvested algae from the reactors (Figure 7a). An oil content similar to that of the seed was measured for the biomass obtained from the first harvest (1H). The oil content percentage was similar for harvests 1H to 4H and slightly increased from harvests 5H to 8H. As stated previously, during this period, the biomass was exposed to low N levels and favorable weather conditions; thus, there were no observed effects from the change in biomass population with regards to oil content (Figure 7a). Even though the oil content ranged between 18% and 22% during this period, the oil productivity increased due to an increase in areal biomass productivity (Figure 4). From harvests 9H to 13H, the oil content decreased most likely due to the increase in N loads; similarly, the biomass and oil productivity decreased. The oil content from the biomass obtained after settling with FeCl_3_ was also evaluated. The results demonstrate that the oil content was not affected by the addition of this coagulant (Figure S5). The only change observed was with respect to the elemental analysis, which contained less carbon compared to the biomass without coagulant. 

On the other hand, the results from both the ER and OR (with harvests every 1–2 weeks or 3–5 days, respectively) indicate that long harvesting intervals favored the accumulation of lipids, whereas increasing the frequency of harvesting aided the biomass areal productivity but not the oil content recovered [45] (Figure 7b). Furthermore, filamentous algae and cyanobacteria (which predominated in OR, as indicated before in Section 3.2.3) had lower lipid content compared to loose microalgae. For instance, [47] reported a lipid content of between 8% and 13% for cyanobacteria, whereas [48] found an oil content of 13.8% in *Stigeoclonium* sp.

### 3.5. Biochemical Methane Potential (BMP) from ER and OR

**Figure 8:**
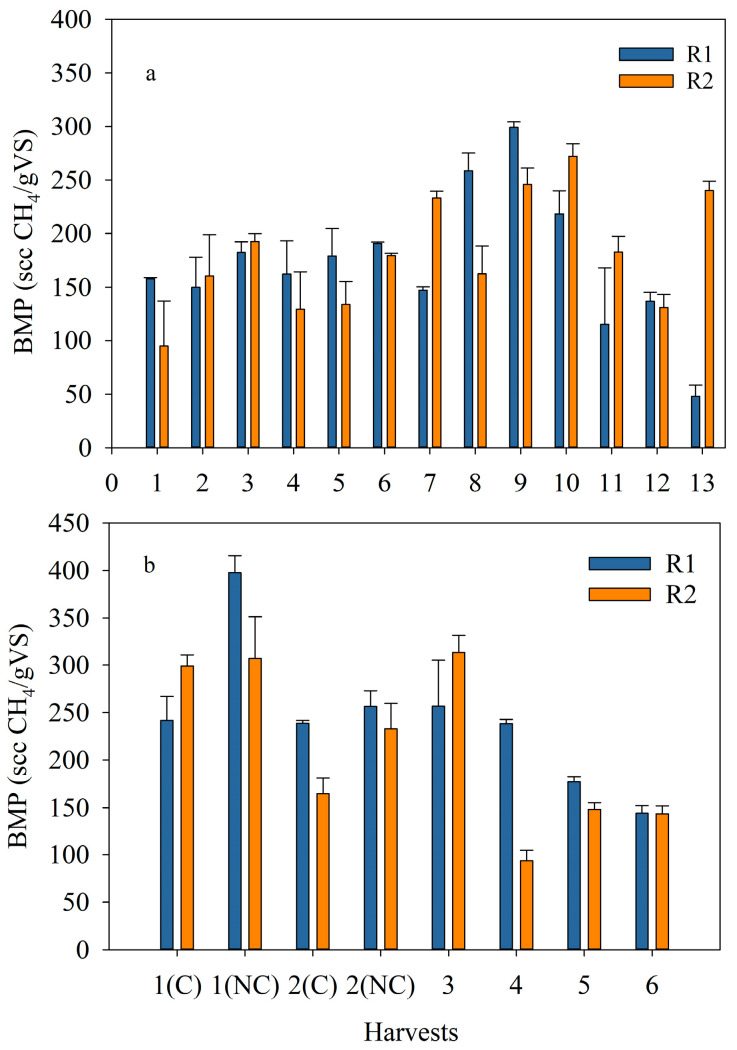
Biochemical methane potential (BMP, scc CH_4_/gVS added) of algae in R1 and R2 along the (**a**) exploratory run and (**b**) optimized run; (C) corresponded to coagulant and (NC) to no coagulant. The data shown are the mean ± error of at least two measurements per reactor per harvest.

Biogas production screening tests on the algal samples were performed with the intent of estimating the biomethane potential (BMP) per gram of the volatile solids (VS) of the algae. The initial *S. obliquus* inoculum was first tested, and a BMP of 105 scc/gVS was obtained. The samples from both runs, ER and OR, were tested for BMP, along with positive (digestion of 1 g of glucose) and negative (digested sludge without any substrate) controls. Theoretically, the biomethane (BMP) that can be produced from the digestion of glucose is 373 scc per gram of substrate, which is similar to the value obtained in this study (i.e., 363.5 scc/g of glucose) (Figure S6). The BMP from the ER, low N feed (1H to 8H), ranged between 150 and 230 scc/gVS, with an evident increase after harvest 8H (Figure 8a). For harvests 9H to 13H, the amount of N used for algae cultivation was increased and the BMP obtained from these harvests ranged from 48 to 300 scc/gVS. Overall, the maximum average BMP was realized from harvest 9H (272 ± 38 scc/gVS), whereas the lowest amount of BMP was obtained from harvest 1H (126 ± 44 scc/gVS), averaging the BMPs produced from the algae harvested from R1 and R2 for each harvesting period. The latter value is similar to BMP results from wastewater-grown *S. obliquus* under similar AD conditions [49].

Figure 8b shows the BMP obtained from each sample digested from the OR while indicating the method used to concentrate the algae. The use of FeCl_3_ did not have a major effect on the BMP. The BMP achieved from this OR varied between 94 and 398 scc/gVS. The maximum average BMP (i.e., 352 ± 62 scc/gVS) was obtained from the first harvest concentrated without the use of coagulant, whereas the lowest average BMP (i.e., 163 ± 21 scc/gVS) corresponded to the last harvest without coagulant. Although the BMP is related to the biochemical composition of the species, it seems that the shift from *S. obliquus* to a consortium did not affect the biogas production. In fact, the BMP obtained was slightly higher for the OR, indicating better digestibility of the filamentous algal polyculture. In addition, the values of BMP obtained in this study are in accordance with the ones obtained by [15] for three single freshwater microalgae: *Chlorella* sp., *Nannochloropsis* sp., and *Scenedesmus* sp.

The elemental analysis performed on the harvested algae from the OR yielded the average percentages by mass of C, H, and N of 45.2%, 6.9%, and 7.7%, respectively, whereas the percentage of O was determined by the difference (40.1%). Based on this elemental analysis, the theoretical BMP realizable from the algae generated from this study was 499 scc/gVS. Compared to the maximum average BMP obtained, this corresponds to about 70% of the theoretical value. As reported in [50], the amount of BMP produced from algae depends on the strain digested, and it typically varies between 24 and 600 scc/gVS. The values of BMP obtained here fall within this range. 

### 3.6. TEA-LCA 

Considering the encouraging results from the pilot system and the data obtained from the optimized run, a technoeconomic analysis (TEA) and a life cycle assessment (LCA) were performed by MicroBio Engineering using a proprietary Environmental Sustainability and Process Economics model based on the CA-GREET model [51] for a full-scale hypothetical 100 ha algae cultivation, harvesting, and processing system for this industrial munitions plant, with an emphasis on maximum biomass production for biogas generation. Two scenarios were investigated: (1) The primary product detailed is raw, unpurified biogas, and (2) raw biogas is upgraded to biomethane with membrane separation. As an additional environmental benefit, coproduct accounting of unrecycled digestate for soil amendment through system boundary expansion was considered in both scenarios. The analysis assumes annual average values for 9 months of facility operation per year (i.e., the algae growing season) and does not provide sensitivity analysis for seasonal variability. The model estimates the costs of the facility using “n-th” plant assumptions, in which the technology is mature, and construction and operation reflects know-how developed from prior facilities, excluding the costly first-of-a-kind facilities, which yields more accurate long-term projections [52]. Furthermore, the construction costs of the ponds and digesters are based on low-cost agricultural engineering practices, recognizing that algae cultivation and digestion are more akin to agricultural rather than manufacturing activity. The assessed algal biomass production facility comprises 100 ha of land, 80% of which would be used for algae cultivation ponds and the remainder for pond berms, buffers, roads, facilities, fencing, etc. The proposed process makes use of CO_2_ from coal-fired flue gas, wastewater, and N from ammonium nitrate wastewater that would otherwise be emitted to the atmosphere or disposed of. The biomass is harvested and thickened via gravity settling to a concentration of approximately 3% volatile suspended solids (VSS). The thickened biomass is then anaerobically digested using in-ground, covered, plug-flow digesters, like those used currently in the dairy and swine industries. The digesters are unmixed and unheated; thus, some natural settling may occur. Up to 90% of the digestate is then recycled back into the production ponds to use bioavailable nutrients such as soluble N and P, whereas the remainder is thickened by polymer addition and screw pressing to use for soil application. The raw biogas can then either be upgraded to biomethane or used on site as raw biogas for processes requiring power and/or heat generation. The TEA process boundary ends after the production of biomethane or biogas. 

As expected, natural gas was less expensive (USD 3.92/MMBTU October 2019) than algae-derived biogas due to the facility’s capital costs. The total cost for raw unpurified biogas was estimated to be USD 43.23/MMBTU. However, the introduction of credits and incentives showed that it may be possible to make this technology competitive if it becomes more heavily incentivized. Although the upgraded biogas scenario involves an additional capital and operating expense for membrane separation, the revenue from a D3 RIN credit (which is not applicable to raw biogas) would considerably the cost, making the estimate decrease to a more affordable and competitive price (USD 5.62/MMBTU). It is important to highlight that this study was performed during the first quarter of 2021 and that nowadays the price of natural gas is higher (USD 8.29/MMBTU in 2022, according to the U.S. Energy Information Agency [53]); therefore, the results should be even more favorable to producing renewable energy.

A life cycle analysis (LCA) was performed for the envisioned algal production facility. The impact category of focus for this LCA was greenhouse gas (GHG) potential, specifically the global warming potential over 100 years (GWP100); thus, a functional unit of grams CO_2_ equivalent per megajoule of energy produced (g CO_2_ eq/MJ) was chosen. Because the LCA does not fully investigate the “cradle-to-grave” inventory of emissions and resource consumption—it excludes biogas transport, power plant resource use, and facility dismantling—the results herein compare emissions on a “well-to-combustion” basis to natural gas extraction, gathering/boosting, and processing. Transportation and power plant costs were assumed to be identical for biogas and natural gas, making them unnecessary to include in the comparison. Additionally, the combustion of biogas was assumed to be a net-zero process because the CO_2_ released is the same CO_2_ captured by the algae biomass. Values for GHG impact were taken from published literature, LCAs involving production of biogas through AD of microalgae, and LCA databases such as Ecoinvent. Furthermore, an expansion of system boundaries was performed in accordance with ISO guidelines to account for impacts of resulting coproducts. From an environmental standpoint, the benefits were large; in fact, net global warming potential (GWP) for scenarios 1 and 2 (i.e., 5.4 g CO_2_ eq/MJ and 13.1 g CO_2_ eq/MJ, respectively) was far more favorable than for the extraction and processing of natural gas (i.e., 104 g CO_2_ eq/MJ). This benefit was largely due to CO_2_ mitigation and the utilization of waste streams as process inputs. 

Overall, the production of biogas obtained from algal biomass is more affordable and competitive than natural fossil gas due to incentive credits. In addition, the environmental impact of producing bioenergy is larger than with natural gas due to the mitigation of CO_2_ and the valorization of waste within the facility.

## 4. Conclusions

The field pilot tests conducted in this study assessed the feasibility of using nitrogen-rich industrial wastewater streams from a munitions production facility to support on-site production of algal biomass under local climatic conditions for the purpose of extracting bioenergy in the form of oil or biogas. This on-site pilot-scale study demonstrated the practicability of the process and the viability of the applied industrial waste stream valorization strategy, which promotes loop closure of nutrients and water resources within the facility and encourages a circular bioeconomy model. 

During the optimized run, during which operational conditions were refined based on the experience gained in the first exploratory run, an average areal biomass productivity of 23.9 ± 0.9 g/m^2^d was obtained during the late spring–early summer testing period. Measurements of oil content and BMP of the harvested biomass indicated that changes in culture conditions and harvesting frequency have a direct impact on the amount of algal oil and biogas produced. Moreover, the values obtained, 20% for oil content and 350 scc/gVS for BMP, are comparable to values reported in the literature, and in the case of biogas, 70% of the theoretical amount was produced. 

In addition, we demonstrated that using a consortium of algae, which is better suited to the local and outdoor conditions and resilient to predators, favored the performance of the process and avoided algae population crashes during the operation. This contributed to improved biomass productivity without vitiating oil or biogas production.

Furthermore, LCA and TEA analyses for a 100 ha algae farm producing biogas were successfully performed with the data obtained in this study. The results illustrate that the price of biogas is affordable and competitive with natural fossil gas due to the incentive credits, while, from an environmental perspective, the benefit of producing bioenergy is larger than with natural gas due to the mitigation of CO_2_ and the valorization of waste within the facility. Lastly, the encouraging results will lead to more actionable research into scale-up to achieve zero waste and, if implemented, indicate potential opportunities for various industrial sectors to reduce industrial waste and lower process costs. 

## Figures and Tables

**Table 1:** Summary of amount and type of nutrients added and harvesting conditions for each test.

Run	N from AN Solution (mg N/L)	Nitrate Supplement (mg N/L)	Sparge CO_2_	Na_2_CO_3_ Addition	Harvest Period	Harvest Included	Volume Recycled (L)
ER—low N	14–30	70	Yes	No	1 or 2 weeks	1H to 8H	0
ER—high N	40–100	70–100	No	Yes	1 or 2 weeks	9H to 13H	0
OR—low N	70–80	0	Yes	No	<1 week (3–5 days)	1H to 7H	400

ER: exploratory run, OR: optimized run, AN: ammonium nitrate filtered solution, H: harvest.

**Table 2:** Characterization of industrial wastewater samples.

Wastewater Name	TC mg C/L	TOC mg C/L	TN mg N/L	NO_3_-N mg N/L	NH_3_-N mg N/L	Amines—N mg N/L	pH
IWWPI	114	49.6	8.65	1.56	7.1	-	6.76
	(77–178)	(15–83)	(4–11)	(0.2–4)	(3.7–11.2)		(6–7)
AN	35,975	29,900	141,705	72,096	38,888	30,721	6.05

TC: total carbon, TOC: total organic carbon, TN: total nitrogen, amines corresponding to monomethylamine and dimethylamine, in brackets range over *n* = 7 samples.

**Table 3:** Toxicological assessment of AN solution based on N concentration.

	Serial Dilutions AN												
TN, mg N/L	50	101	126	202	252	303	403	504	756	1008	1523	2017	
NH_4_-N, mg N/L	16	31	39	62	78	93	124	156	233	311	467	622	
NO_3_^−^-N, mg N/L	23	45	56	90	113	135	181	226	339	452	677	903	
Amines—N, mg N/L	12	25	31	49	61	74	98	123	184	246	369	492	
µ, d^−1^	0.75	0.73	0.70	0.73	0.69	0.71	0.68	0.68	0.68	0.62	0	0	
Inh, %	0	0.83	3.45	0.82	4.55	2.51	6.78	6.92	5.28	14.27	100	100	

**Table 4:** Temperature (°C) inside both reactors and throughout the seasons during the exploratory run.

	Total Period	Spring	Summer	Fall
Average temperature	26.8 ± 0.3	26.0 ± 0.3	28.8 ± 0.2	23.6 ± 0.2
Max day temperature	50.0 ± 0.4	39 ± 3	50.0 ± 0.4	44 ± 3
Min night temperature	12.4 ± 0.4	15.3 ± 0.4	19.9 ± 0.4	12.4 ± 0.4

## Data Availability

All data generated or analyzed during this study are included in the published article.

## Supplementary Materials

The following supporting information can be downloaded at: https://www.mdpi.com/article/10.3390/bioengineering10121339/s1, Figure S1: Toxicity assessment for N species in AN wastewater stream (top) and image of the 24-well microplate at the end of the test (bottom). Values correspond to N in mg/L. C correspond to control; Figure S2. Microalgae and cyanobacteria identified by shotgun metagenomic sequencing; Figure S3. Correlation between dry biomass (AFDW, g/L) and fluorescent units (FU); Figure S4. Mass spectrometry scans from biomass extracts in negative (left) and positive (right) modes; Figure S5. Variation in the oil content of algae after harvesting and concentration; Figure S6. Plot of cumulative biomethane production for positive control (glucose) and a sample (digested algae) versus number of days for digestion.
