# Supplementary material for: On-Site Pilot-Scale Microalgae Cultivation Using Industrial Wastewater for Bioenergy Production: A Case Study towards Circular Bioeconomy
Source: Bioengineering (Basel). 2023 Nov 21;10(12):1339. doi: 10.3390/bioengineering10121339 (PMC10740855; doi:10.3390/bioengineering10121339)
Supplement: Supplementary file 1 [file bioengineering-10-01339-s001.zip › bioengineering-2707577-supplementary.pdf]

bioengineering

Supplementary Materials

On-Site Pilot-Scale Microalgae Cultivation Using Industrial Wastewater for Bioenergy Production: A Case Study towards Circular Bioeconomy

Juliana Abraham [1,*], Tobi Abimbola [2], Washington J. Braida [1], Amalia Terracciano [1], Tsan-Liang Su [1], Christos Christodoulatos [1], Agamemnon Koutsospyros [1], Abhishek RoyChowdhury [1], Benjamin Smolinski [3] and Adeniyi Lawal [2]

[1] Center for Environmental Systems, Stevens Institute of Technology, Hoboken, NJ 07030, USA; wbraida@stevens.edu (W.J.B.); aterrac1@stevens.edu (A.T.); t1su@stevens.edu (T.-L.S.); christod@stevens.edu (C.C.); akoutsos@stevens.edu (A.K.); aroychowdhury@navajotech.edu (A.R.)

[2] New Jersey Center for Microchemical Systems , Department of Chemical Engineering and Materials Science, , Stevens Institute of Technology, Hoboken, NJ 07030, USA; tabimbol@stevens.edu (T.A.); alawal@stevens.edu (A.L.)

[3] Combat Capabilities Development Command—Armaments Center (DEVCOM—AC), Picatinny Arsenal, Dover, NJ 07806, USA; benjamin.l.smolinski.civ@army.mil

* Correspondence: jabraham@stevens.edu

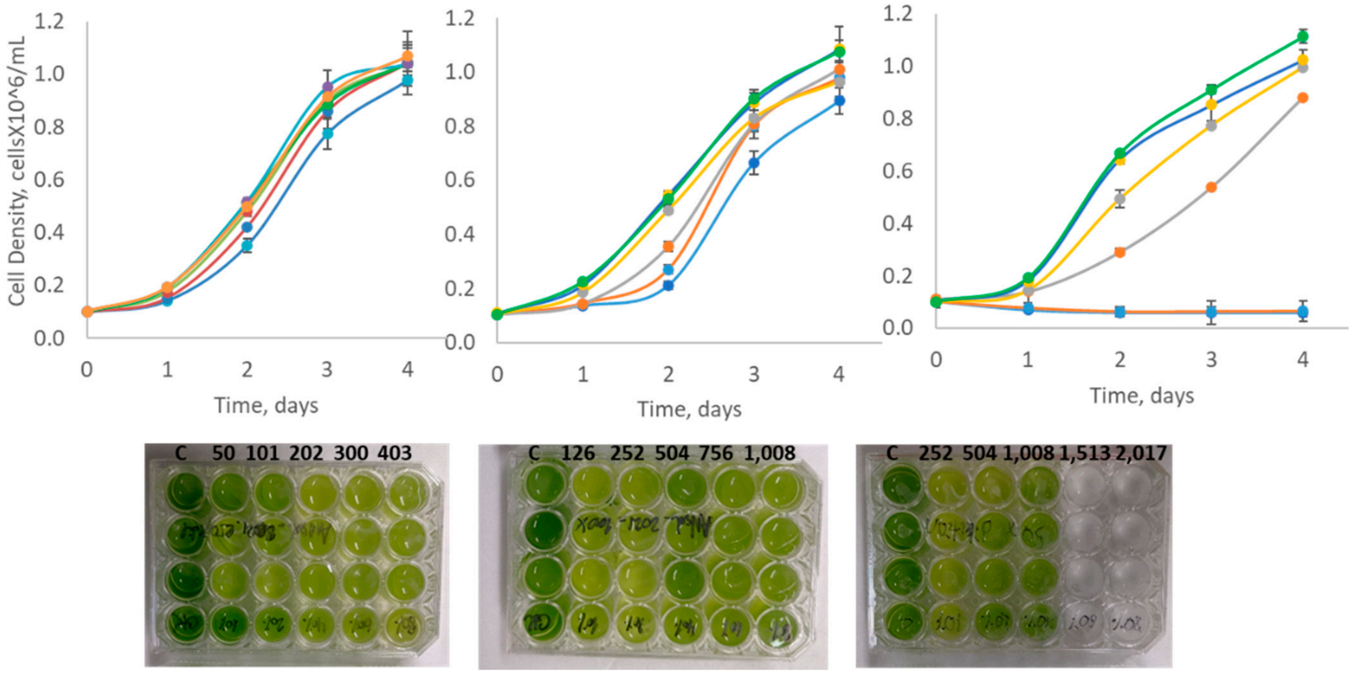

Figure S1. Toxicity assessment for N species in AN wastewater stream (top) and image of the 24-well microplate at the end of the test (bottom). Values correspond to N in mg/L. C correspond to control (dark green line).

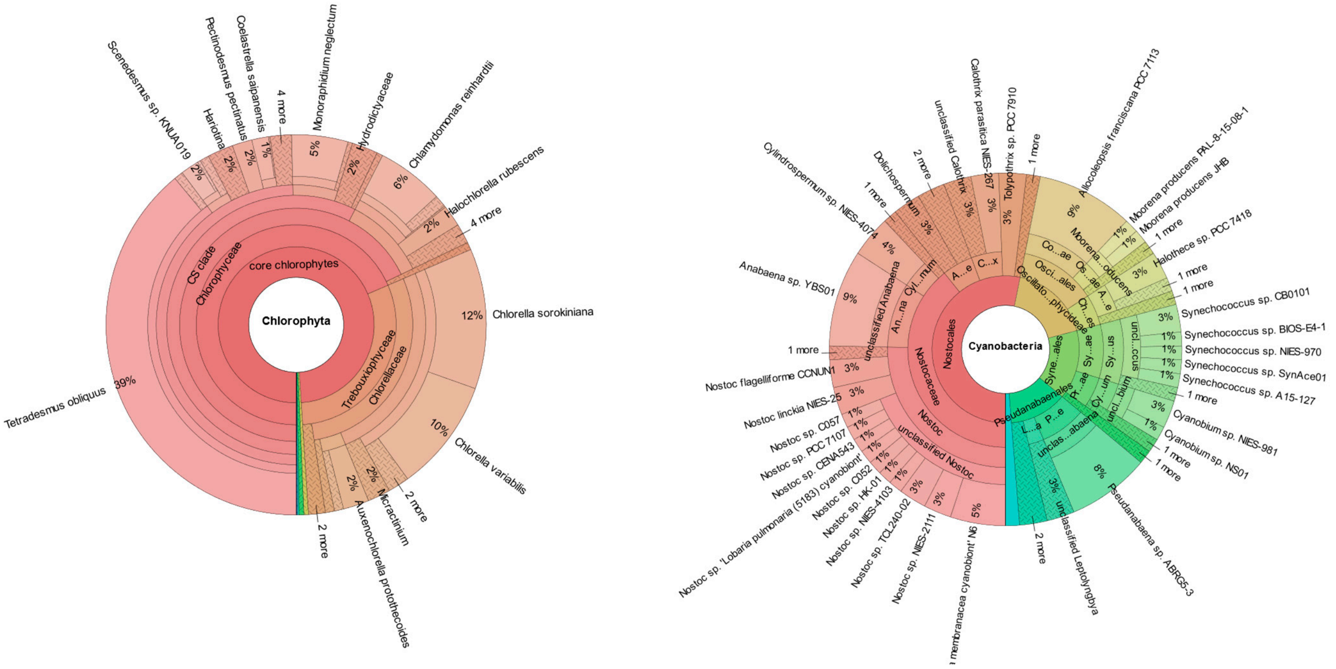

Figure S2. Microalgae and cyanobacteria identified by shotgun metagenomic sequencing.

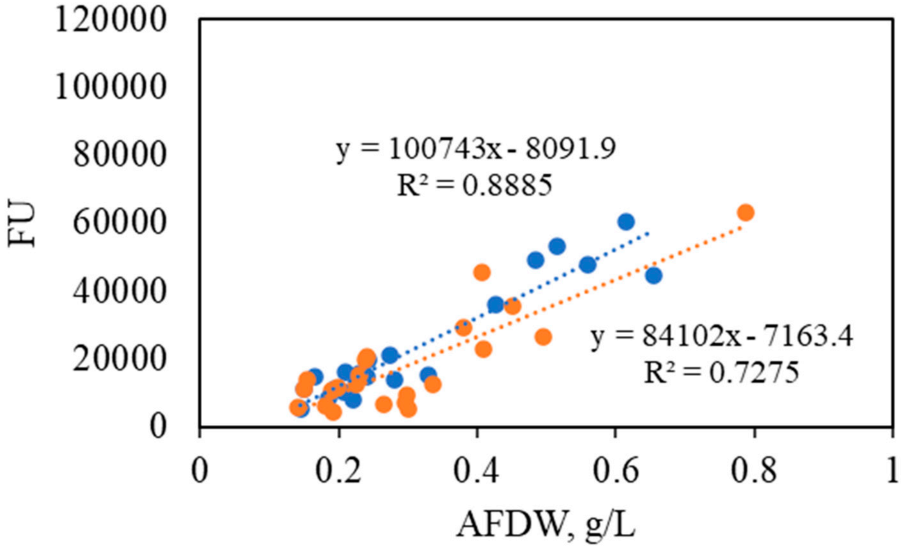

Figure S3. Correlation between dry biomass (AFDW, g/L) and fluorescent units (FU).

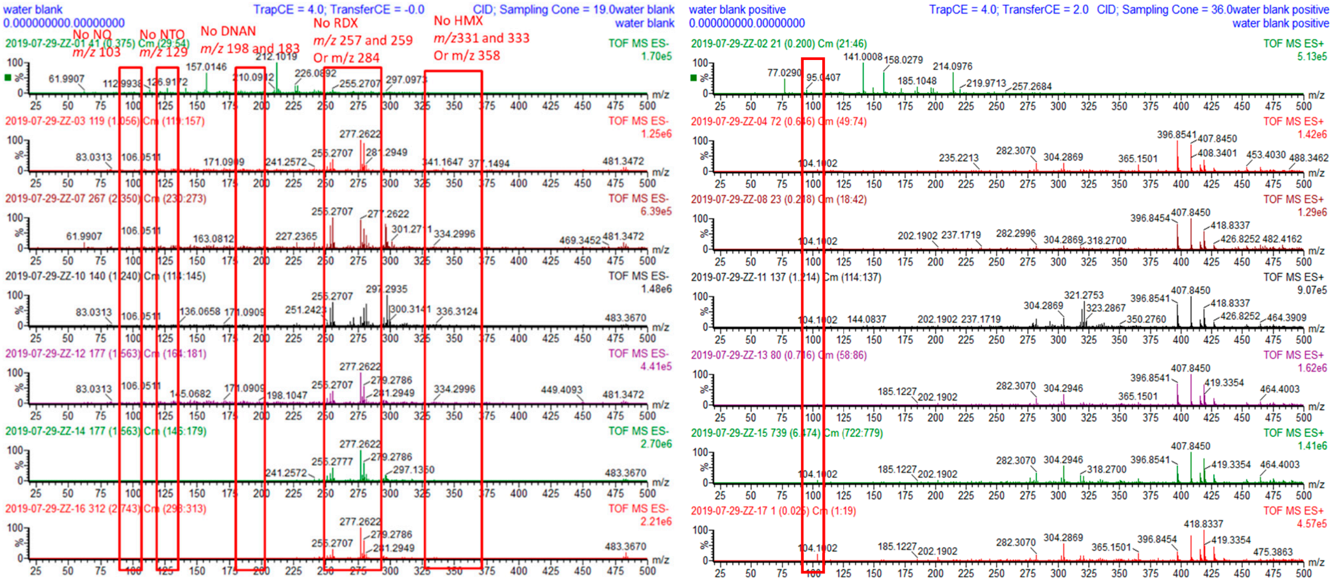

Figure S4. Mass spectrometry scans from biomass extracts in negative (left) and positive (right) modes.

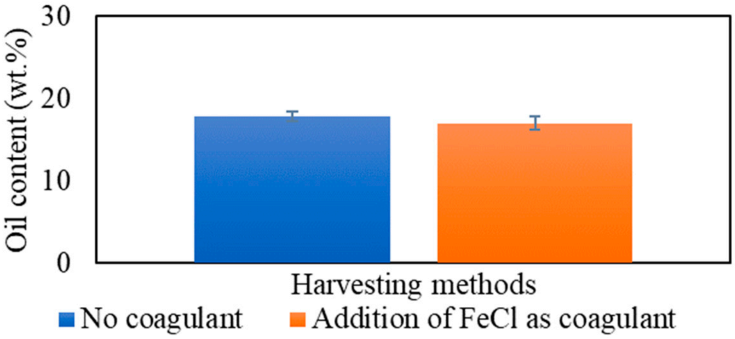

Figure S5. Variation in the oil content of algae after harvesting and concentration.

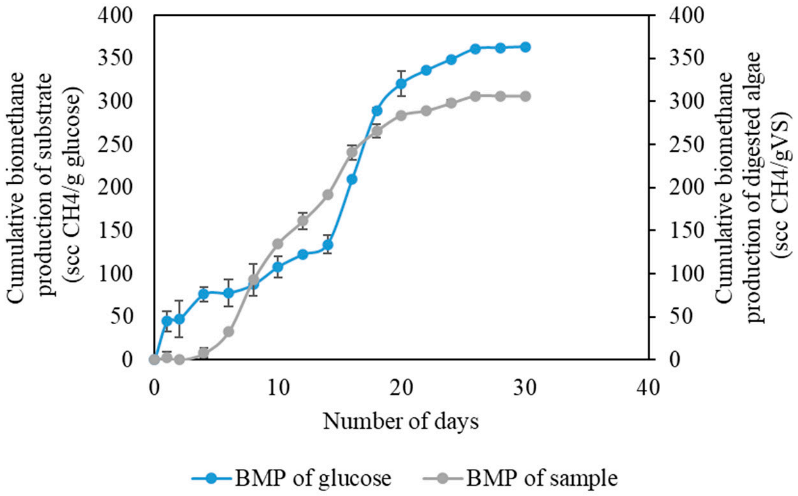

Figure S6. Plot of cumulative biomethane production for positive control (glucose) and a sample (digested algae) versus number of days for digestion.
